# Drug Delivery from Stimuli-Responsive Poly(N-isopropylacrylamide-co-N-isopropylmethacrylamide)/Chitosan Core/Shell Nanohydrogels

**DOI:** 10.3390/polym14030522

**Published:** 2022-01-27

**Authors:** Andrés Ortega-García, Bryan Giovanny Martínez-Bernal, Israel Ceja, Eduardo Mendizábal, Jorge Emilio Puig-Arévalo, Lourdes Adriana Pérez-Carrillo

**Affiliations:** 1 Chemical Engineering Department, University Center of Exact Sciences and Engineering (CUCEI), University of Guadalajara (UdG), Guadalajara 44100, Jalisco, Mexico; a_ortegag@yahoo.com.mx (A.O.-G.); bryan.martinez5476@alumnos.udg.mx (B.G.M.-B.); puig_jorge@hotmail.com (J.E.P.-A.); 2 Physics Department, University Center of Exact Sciences and Engineering (CUCEI), University of Guadalajara, Guadalajara (UdG), Guadalajara 44100, Jalisco, Mexico; israel.ceja@academicos.udg.mx; 3 Chemistry Department, University Center of Exact Sciences and Engineering (CUCEI), University of Guadalajara (UdG), Guadalajara 44100, Jalisco, Mexico; eduardo.mmijares@academicos.udg.mx

**Keywords:** nanohydrogels, stimuli-responsive polymers, core-shell polymers, drug delivery

## Abstract

The synthesis of stimulus-responsive poly(N-isopropylacrylamide-co-N-isopropylmethacrylamide)/chitosan core/shell nanohydrogels made by batch emulsion polymerization in the presence of chitosan (CS) micelles is reported. The ratio of monomers required to obtain copolymers with a volume phase transition temperature (T_VPT_) in the range of the temperatures observed in the human body in response to an infection (38 to 40 °C) was estimated with the Fox equation. The conversion was determined by gravimetry; mean particle size, size distribution, and thermal response were measured by quasi-elastic light scattering (QLS). The core/shell structure was confirmed by TEM, and FTIR showed the presence of N-isopropyl acrilamide (NIPA), N-isopropyl methacrylamide (NIPMA), and CS in the nanohydrogels. The nanohydrogels were loaded with the drug doxycycline hyclate, and their release kinetic profile was determined at pH = 2.0 and 7.4 at their volume phase transition temperatures (T_VPT_). A higher amount of drug was released at acidic pH. Some mathematical models described in the literature were used to fit the experimental drug release data.

## 1. Introduction

Stimuli-responsive polymers are materials that have the ability to significantly change a property (or properties) in response to external environmental conditions [1,2]. Typical stimuli are temperature, pH, ionic strength, magnetic and electrical fields, and light and redox potential [3]. Stimuli-responsive polymers can be designed to act as biosensors [4] in ion-exchange chromatography [5], in micromechanical materials [6], in chemotherapy [7,8], in drug delivery [9,10], in photo-responsive coating materials [11] and in photonics [12].

Stimuli-responsive nanoparticles have been used recently as drug carriers to deliver active ingredients such as drugs, peptides, and genes [13]. Because there are specific conditions (temperature and pH, among others) to which stimuli-responsive nanoparticles loaded with specific drugs can respond, the importance of these nanoparticles is evident, particularly when they respond to more than one stimulus simultaneously [14]. Hyperthermia commonly occurs in diseased tissues compared to normal tissues, so several temperature-sensitive nanohydrogels have been developed for drug delivery purposes [15,16]. 

The most used thermosensitive polymer is poly(N-isopropylacrylamide) (PNIPA) which has a lower critical solution temperature (LCST) or volume phase transition temperature (T_VPT_) at 32 °C [17]. Below 32 °C, PNIPA is water soluble, but above this temperature it becomes insoluble [18]. This property is due to the polymer’s ability to change from hydrophilic to hydrophobic due to conformation changes from globules to coils where the surrounding temperature exceeds its LCST [14]. Poly(n-isopropylmethacrylamide) (PNIPMA) is also a thermosensitive polymer and has a T_VPT_ of 46 °C [19,20,21]. Therefore, polymerization of a mixture of these two monomers should produce a polymer with T_VPT_ between 32 and 46 °C. M. Kokufuta, S. Sato and E. Kokufuta [22] reported the T_VPT_ behavior of copolymers of NIPA and NIPMA employing a combination of turbidity measurements and Differential Scanning Calorimetry (DSC) [22]. These authors found that the temperature at which these copolymers exhibit T_VPT’S_ increased linearly with the increase of NIPMA mole fraction in the copolymer. 

CS is a pH-responsive material that can form micelles in an acetic acid aqueous solution [23] and because of its good biocompatibility, biodegradability and non-toxicity is a promising material for drug delivery and other pharmaceutical applications [24]. Huang et al. reported obtaining pH- and temperature-sensitive polyNIPA/CS core/shell nanoparticles which they used for drug release tests [25].

This work reports the synthesis of temperature- and pH-responsive poly(NIPA-co-NIPMA)/CS core/shell nanohydrogels. The ratio of monomers required to obtain copolymers with a T_VPT_ in the range of 38 to 40 °C was estimated with the Fox equation. Core/shell nanohydrogels were prepared by emulsion polymerization in the presence of CS micelles. The nanohydrogels were loaded with the drug doxycycline hyclate, and their release kinetic profile was determined at pH = 2.0 and 7.4 at their volume phase transition temperatures (T_VPT_) and compared with the predictions of models commonly used in drug release studies.

## 2. Materials and Methods

NIPA, with a purity of 99%, was from Acros Organics (Waltham, MA, USA). Chitosan (CS) with a degree of de-acetylation of 68% [26]; NIPMA, 97% pure; the crosslinker N, N′-methylenbisacrylamide (NMBA), 99% pure; Brij 58, employed as the surfactant, and glutaraldehyde ((GA), 50% in water) were all from Sigma-Aldrich. The initiator, potassium persulfate (KPS), was ACS grade from Fermont. N_2_ gas was from Infra de Occidente (Guadalajara, Mexico), and the double-distilled water came from Productos Selectropura (Mexico). The model drug selected for the delivery studies from the core/shell nanoparticles was doxycycline hyclate from Sigma-Aldrich (St. Louis, MO, USA).

First, poly(NIPA-co-NIPMA) hydrogels with various weight ratios of NIPA/NIPMA and NMBA as crosslinking agent were synthesized by batch emulsion polymerization using Brij 58 as the surfactant. To reduce the number of experiments to determine the weight ratio of NIPA and NIPMA that would allow obtaining the required collapsing temperatures (38, 39 and 40 °C), an estimate of the weight ratio was obtained with the Fox equation employing a T_VPT_ value of 32 °C for the PNIPA and 46 °C for the PNIPMA. The Fox equation has the form [27]:

$$\frac{1}{T_{\mathrm{VPTc}}}=\frac{X_{1}}{T_{\mathrm{VPT}1}}+\frac{X_{2}}{T_{\mathrm{VPT}2}}$$

where T_VPT1_ and T_VPT2_ refer to PNIPA and PNIPMA, respectively, while T_VPTc_ is the sought after temperature (38, 39, or 40 °C), and *X_1_* and *X_2_* are the mole fractions of NIPA and NIPMA. Table 1 shows the weight ratios of NIPA and NIPMA required to produce the desired T_VPT_ and the moles of monomer added to the reactor. 

The poly(NIPA-co-NIPMA) core/shell nanohydrogels were synthesized by batch emulsion polymerization. First, in a 250 mL glass reactor, 0.667 g of CS was dissolved in 48.114 g of 1 wt % acetic acid solution. The reactor was kept with continuous agitation under continuous N_2_ bubbling. Then the moles of NIPA and NIPMA determined by the Fox equation to produce the T_VPT’S_ (38, 39, or 40 °C), and the NMBA were added (Table 1). To initiate the reaction, 0.099 g of KPS dissolved in 3.0 g of water were added. The reaction was carried out at 70 °C for 120 min. Then the temperature was decreased to 40 °C, and 0.40 g of GA aqueous solution was added to crosslink the chitosan shell. The reaction was allowed to continue for one hour. A sample of the final latex was cooled to 25 °C, placed in a dialysis bag (*Mn* = 11.2 kg/mol exclusion size), and immersed in distilled water under sink conditions for 72 h to remove residual monomers initiator and unreacted GA. Distilled water was replaced every 24 h. The dialyzed sample was dried in a convection oven at 60 °C to obtain constant weight, and the final conversion was determined with the following formula [15]:

$$X=\frac{W_{dry}-W_{CS}-W_{GA}}{W_{NIPA}+W_{NMBA}}\;\;\;\;\;$$

where *W_dry_*, *W_CS_*, *W_GA_*, *W_NIPA_*, and *W_NMBA_* are the masses of the dry sample, chitosan, glutaraldehyde, *NIPA*, and *NMBA*, respectively.

To measure particle size and size distribution, samples were taken at the end of the copolymerization reaction and after the glutaraldehyde was crosslinked. The particle size and size distribution were determined with a Nanosize S-90 Quasielastic Light Scattering (QLS) apparatus from Malvern instruments. For the measurements, 0.05 g of each copolymer/CS latex was dispersed in 10 g of water. From these dispersions, 3 mL was placed in a QLS quartz cell for the measurements. To determine the T_VPT_ of the copolymers obtained, they were heated from 25 to 45 °C with increments of 5 °C, allowing 15 min before doing the next temperature increment. Measurements were made at least three times for each sample.

The core-shell nanohydrogels were examined in a Nicolet iS5 FTIR spectrophotometer to verify the presence of poly(NIPA-co-NIPMA) and CS. The size, shape, and morphology of the poly(NIPA-co-NIPMA)/CS core/shell nanoparticles were determined in a JEOL 1200 EXII Transmission Electron Microscope (TEM). To perform this, one drop of the latex was diluted 20 times with water, deposited in a grid, which was dried overnight before observation in the TEM.

For the drug loading, 0.201 g of poly(NIPA-co-NIPMA)/CS core/shell nanoparticles was dispersed in 80 g of water containing 0.021 g of doxycycline hyclate. This dispersion was maintained at constant agitation at pH of 4.5 and 25 °C during 72 h. The pH of the dispersion was then adjusted to a pH of 9.0, and the drug-loaded nanoparticles were separated by centrifugation at 13,500 rpm for 5 min. Then the nanoparticles were dried by lyophilization and weighed. Three samples of the supernatant layer were taken to determine their drug content with a Thermoelectron UV-vis spectrophotometer (Genesis 10 UV) at a wavelength of 346 nm and a doxycycline hyclate calibration curve at pH = 9.0. The drug in the nanoparticles was obtained by subtracting the drug in the supernatant layer from the amount of drug loaded. 

For the drug release tests, solutions were prepared by mixing 5.4 g of NaCl and 600 mL of a buffer solution at pH = 2.0 or 7.4. Then 50 g of this saline solution was placed in a glass bottle and put in a water bath at the desired temperature (38, 39, or 40 °C). The flask was left for 5 min to ensure that the solution attained the desired temperature. Then, 25 mg of drug-loaded nanoparticles was put in a dialysis bag containing 1 mL of the saline solution at the desired pH (2.0 or 7.4), and the bag was placed inside the glass bottle. For each measurement, 0.5 mL of saline solution was withdrawn from the bottle for analysis, and 0.5 mL of saline solution of the same pH of the sample taken was replaced in the bottle to maintain sink conditions. To determine drug content, the withdrawn solutions were placed in a quartz cell, weighed (*W_ML_*), diluted with a saline solution at the pH of the sample taken, and then re-weighed (*W_TL_*). The diluted samples were analyzed using the UV-vis spectrometer at a wavelength of 346 nm. With the absorbance values (*A*), the concentration of the drug in solution (*M_L_*) was determined using the calibration curve at the system’s pH. The amount of drug released, *F*(mg), and its percentage, *%F_L_*, were calculated according to the following formulas:

$$\left(a\right)\;\;F\left(\mathrm{mg}\right)=\frac{M_{L}\times W_{TL}}{W_{ML}}\times W_{MSS}\;\;\;\;\;\;\;\;\left(b\right)\;\;\%F_{L}=\frac{F\left(\mathrm{mg}\right)}{F_{NP}}\times 10$$

## 3. Results

Table 2 shows that high conversions were obtained and that the average particle size of the nanogels increased after de chitosan was crosslinked. 

**Figure 1:**
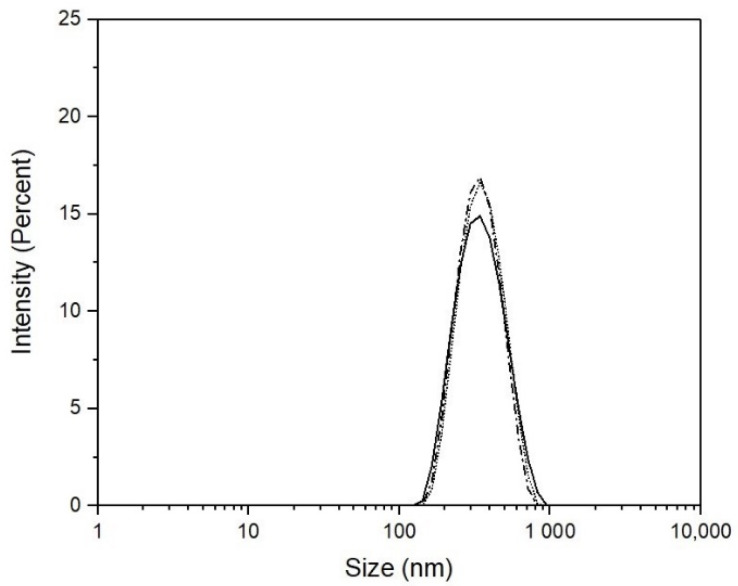
QLS-intensity of the particle size distributions at 25 °C. T_VPT_ of 38 °C (―); T_VPT_ of 39 °C (– –); T_VPT_ of 40 °C (⋯).

Figure 1 shows QLS-intensity particle size distributions at 25 °C of the three poly(NIPA-NIPMA)/CS core/shell nanohydrogels with T_VPT_ of 38, 39, and 40 °C. The distributions are unimodal and similar.

**Figure 2:**
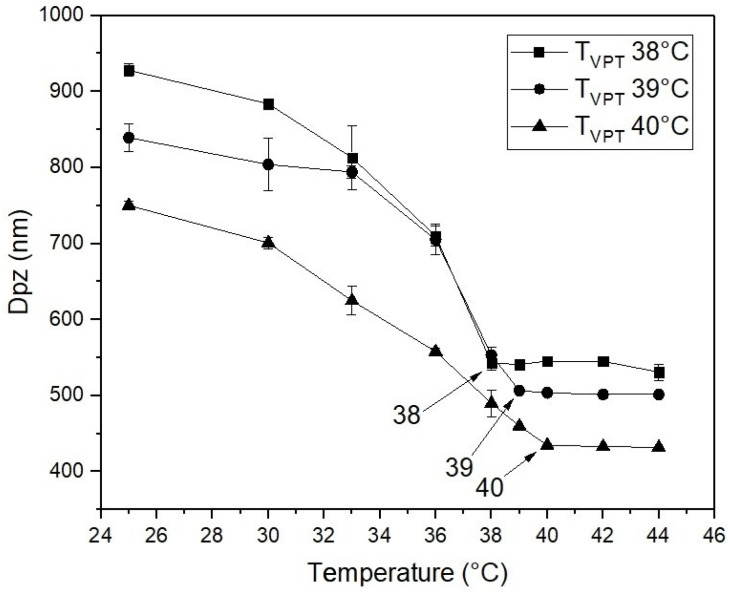
QLS-intensity particles diameter versus temperature for the poly(NIPA-co-NIPMA) hydrogels made by emulsion polymerization with T_VPT_ of 38 °C (◼); T_VPT_ of 39 °C (●); T_VPT_ of 40 °C (▲).

Figure 2 shows the plots of QLS intensity particle diameter as a function of temperature for the three poly(NIPA-co-NIPMA) nanohydrogels fabricated by batch emulsion polymerization with T_VPT_ of 38, 39, and 40 °C. In this Figure it can be observed that particle size of the poly(NIPA-co-NIPMA) hydrogels decreases with increasing temperature up to their respective T_VPT_; the particle diameter remains almost constant at a temperature above the collapse temperature. When the NIPMA/NIPA ratio increases, the particle size decreases.

**Figure 3:**
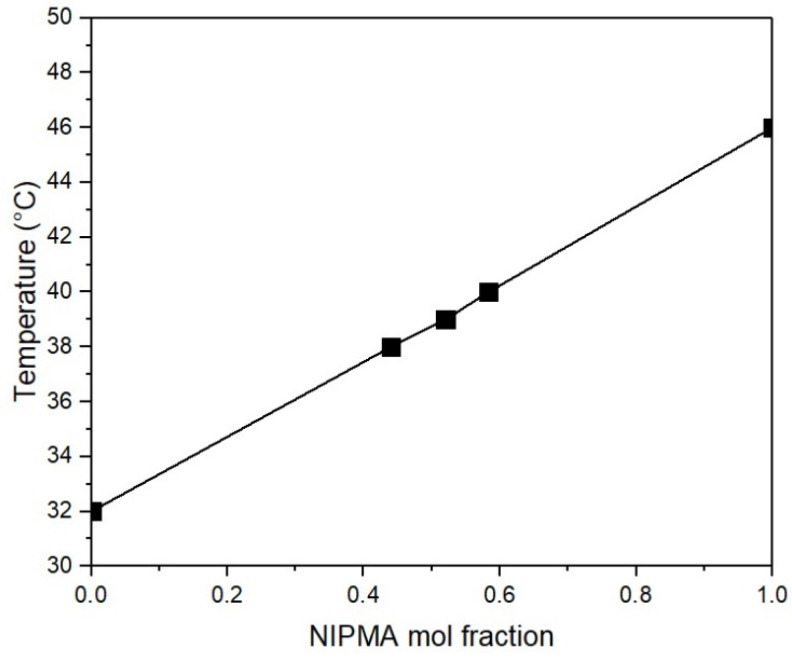
Effect of PNIPMA mole fraction on the T_VPT_ of the poly(NIPA-NIPMA) hydrogels.

Figure 3 shows a linear increase in T_VPT_ with increasing NIMPA mole fraction. This result is similar to that reported in the copolymerization of NIPA and NIPMA in water [22].

**Figure 4:**
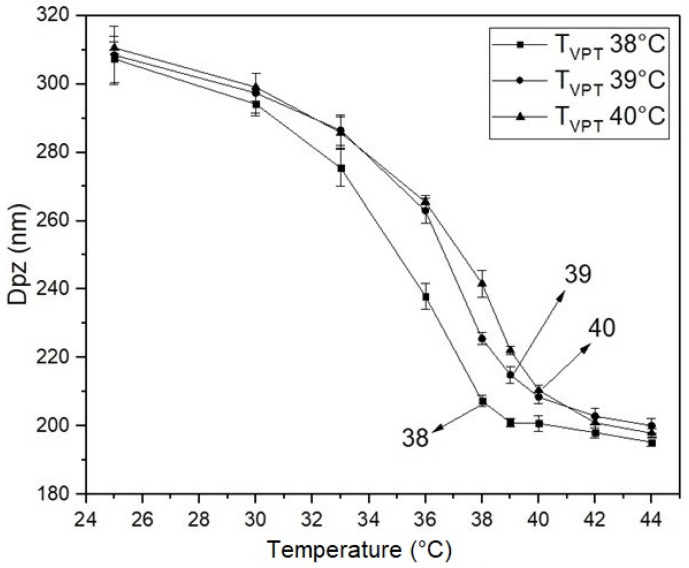
QLS-intensity nanohydrogels diameter versus temperature for the three poly(NIPA-co-NIPMA)/CS nanohydrogels made by batch emulsion polymerization: T_VPT_ of 38 °C (◼); T_VPT_ of 39 °C (●); T_VPT_ of 40 °C (▲).

Figure 4 displays plots of particle diameter as a function of temperature for the poly(NIPA-co-NIPMA)/CS core/shell nanohydrogels. Particle size decreases as temperature increases up to their respective T_VPT_; above the collapse temperature, particle diameter decreases slightly. 

**Figure 5:**
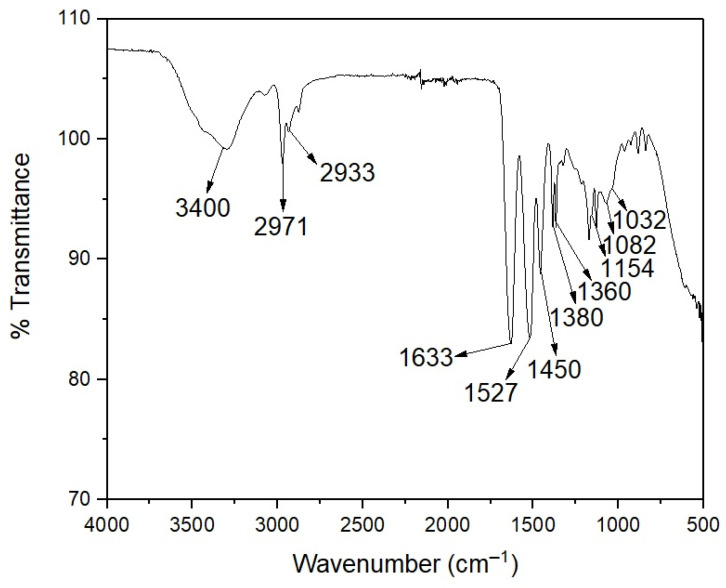
FTIR spectrum of the poly(NIPA-co-NIPMA)/CS core/shell nanohydrogels with a T_VPT_ of 40 °C.

Figure 5 shows the FTIR spectrum of the poly(NIPA-NIPMA)/CS core/shell nanohydrogels. A broad peak around 3400 cm^−1^ is due to the N–H stretching vibration of the amide groups of poly(NIPA-NIPMA) and the amino group of CS. The bands at 1360 and 1380 cm^−1^ are due to the isopropyl group. The band at 1527 cm^−1^ is due to the bending vibration of the amide groups, and the peak from the stretching vibration at 1633 cm^−1^ of the C=O group corresponds to the nanohydrogel [28]. This Figure also shows the pyranose ring bands at 1082 and 1032 cm^−1^ of the CS six-membered ring and the peak at 1450 cm^−1^ of the C–O–C asymmetric stretching band [29].

**Figure 6:**
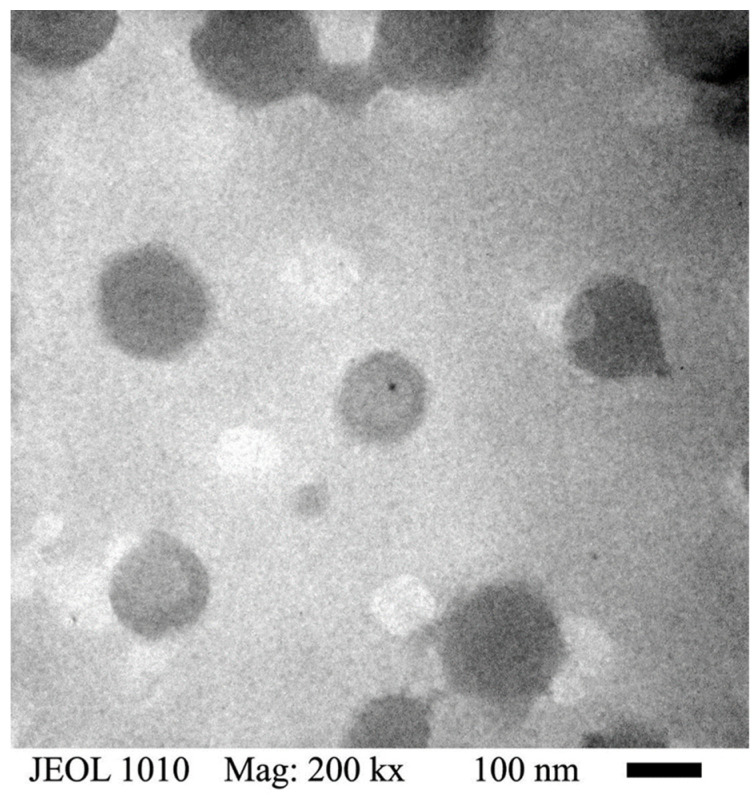
TEM micrograph of the poly(NIPA-co-NIPMA)/CS core/shell nanohydrogels with T_VPT_ of 39 °C.

Figure 6 shows a TEM micrograph of the poly(NIPA-co-NIPMA)/CS nanohydrogels with T_VPT_ of 39 °C; the micrograph reveals that they are spherical with average sizes in the range of 120 to 150 nm. This micrograph shows that the nanoparticles have a dark core and a thin, lighter shell. Micrographs of nanohydrogels with T_VPT_ at 38 and 40 °C show similar particle sizes.

Table 3 shows the percentage of drugs loaded in the core/shell nanohydrogels.

**Figure 7:**
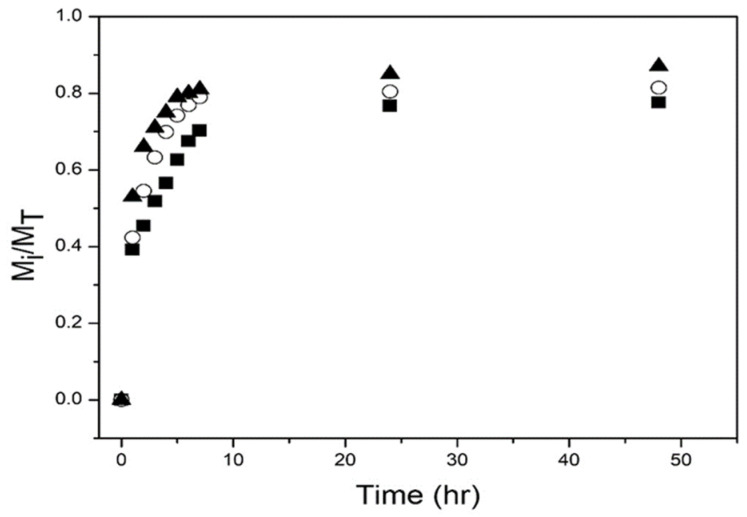
Experimental drug release data for poly(NIPA-co-NIPMA)/Chitosan core/shell nanohydrogels at pH = 2. T_VPT_ of 38 °C (◼); T_VPT_ of 39 °C (ο); T_VPT_ of 40 °C (▲).

Figure 7 shows the drug release profiles from the nanohydrogels at pH = 2.0. During the first three hours, release rates are fast, then start to decrease; after 50 h, the amount of drug released was approximately 77% to 87%. A slighter higher amount of drug is released as the NIPMA/NIPA ratio increased.

**Figure 8:**
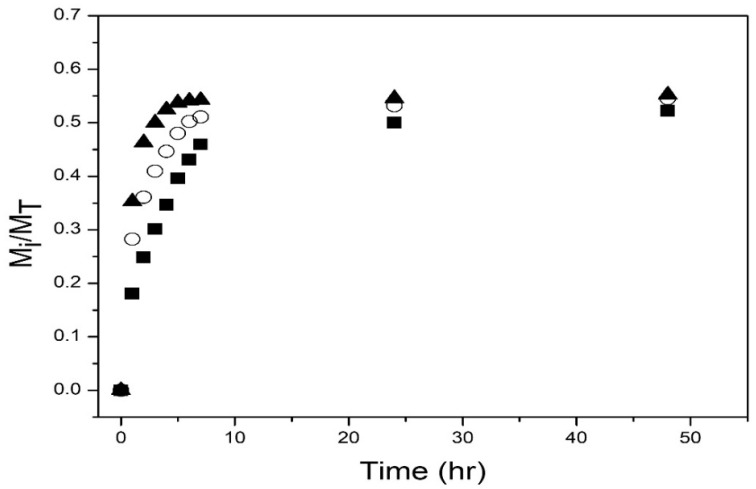
Experimental drug release data for poly(NIPA-co-NIPMA)/Chitosan core/shell nanohydrogels at pH = 7.4: T_VPT_ of 38 °C (◼); TVPT of 39 °C (ο); TVPT of 40 °C (▲).

Figure 8 depicts the drug release profiles at pH = 7.4 of the three nanohydrogels. Again, the amount released increases rapidly during the first 3 h, then the release rate decreases; after 50 h, the amount of drug released is ca. 53% to 55%. However, the amounts released are much lower than those obtained at pH = 2 (see Figure 7).

Several mathematical models published in the literature were used to fit the experimental release data to determine the drug release mechanism [30].

The Korsmeyer–Peppas model is the most widely used for fitting drug release data. It is based on a diffusion mechanism and is a modification of the Higuchi model [31,32].

$$\frac{M_{i}}{M_{T}}=k\times t^{n}$$

*M_i_* is the amount of drug released at time *t*, *M_T_* is the total amount of drug loaded, *k* is the Korsmeyer rate constant, and *n* is the release exponent indicating the release mechanism. 

The extended Korsmeyer–Peppas model is used when initially there is a rapid drug release “the burst effect” and is obtained by adding a constant term “*b*” to the Korsmeyer–Peppas model [33,34].

$$\frac{M_{i}}{M_{T}}=b+k\times t^{n}\;$$

The Weibull equation can be applied to almost all types of dissolution curves. Here *k_w_* is the Weibull constant related to the inverse of the time required to release 63.2% of the drug, and *n* indicates the shape of the curve [35,36].

$$\frac{M_{i}}{M_{T}}=1-e^{{\left(-\frac{t}{kw}\right)}^{n}}$$

The first-order model, derived by Noyes–Whitney, predicts a first-order dissolution process. However, when the asymptotic value released from the drug is less than one, an additional parameter “*k*” is added to obtain a better fit for the release data [34,35].

$$\frac{M_{i}}{M_{T}}=k\left(1-e^{-nt}\right)$$

where *n* is the kinetic constant.

The Hixson–Crowell model assumes the dissolution rate is a function of the cube root of the particle volume and that the particle radius is not constant [35].

$$M_{T}^{1/3}-M_{R}^{1/3}=k_{H}\times t$$

*M_R_* is the remaining amount of drug in the particles at time *t*, and *k_H_* is the Hixson–Crowell constant.

**Figure 9:**
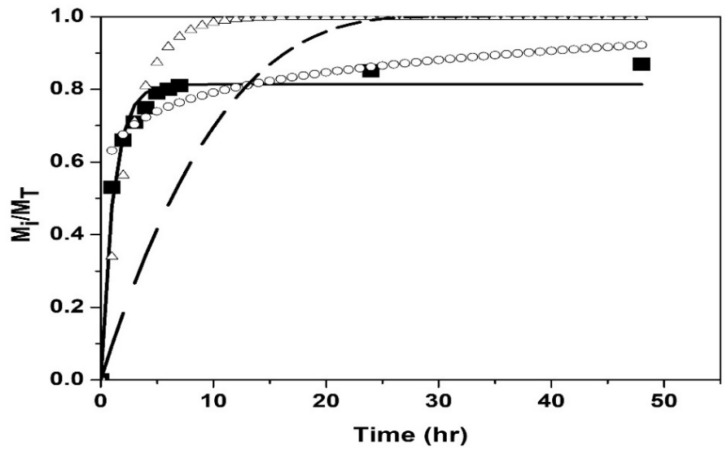
Comparison of the models with the experimental drug release data of using nanohydrogels with T_VPT_ of 40 °C at pH = 2.0: Experimental (◼); Weibull (∆); Korsmeyer–Peppas and extended Korsmeyer–Peppas (ο); first order (▬); Hixson–Crowell model (─ ─).

The drug release kinetic profile was determined at pH = 2.0 and 7.4 at their volume phase transition temperatures (T_VPT_). Figure 9 andFigure 10 show the fit of the models to the experimental data of drug release from the poly(NIPA-co-NIPMA)/CS nanohydrogels with a T_VPT_ of 40 °C, at pH = 2.0 and 7.4, respectively.

Table 4 shows the fit parameters and the determination coefficient, *R^2^*, of the models used. 

## 4. Discussion and Conclusions

Drug delivery from stimuli-responsive nanoparticles, particularly those that respond simultaneously to temperature and pH, have received increased attention in recent years [14,37]. Fundenau et al. [38] reported that the poly(NIPA-co-NIPMA) with a 51/49 weight ratio NIPA/NIPAM displays an LCST of 36.8 °C at pH = 7.4 employing a phosphate buffer solution. The microgels were loaded with dexamethasone, and their release at temperatures below and above LCST was investigated. Huang et al. obtained pH- and temperature-sensitive PNIPA/CS core/shell nanoparticles by emulsion polymerization with a T_VPT_ of 33.4 °C [25]. Alvarado et al. reported the synthesis of PNIPA/CS core/shell nanoparticles by semi-continuous heterophase polymerization (SHP) with a T_VPT_ of 34 °C [26]. However, the T_VPT_ of these nanoparticles is lower than the temperatures in the human body in response to infection.

Here we report the synthesis and characterization of emulsion polymerization of core/shell nanoparticles composed of poly(NIPA-co-NIPMA) in the core and CS forming the shell with T_VPTs_ of 38, 39, and 40 °C, for efficient drug delivery upon infection. 

First, copolymers of NIPA and NIPMA were synthesized by emulsion polymerization using Brij 58 as a surfactant. The weight ratios of NIPA to NIPMA to be used in the synthesis were estimated with the Fox equation. The weight ratio predicted with this equation produced copolymers with T_VPT_ close to the desired values as shown in Figure 2. This result can be explained because the Fox equation [27] was developed for molecules with similar structures and solubility parameters (similar cohesive energy density), which is the case for NIPA and NIPMA molecules. Figure 2 shows that the diameter of the nanohydrogels determined by QLS decreased with increasing temperature due to water expulsion and copolymer collapse. When the temperature was higher than its T_VPT_, the diameter remained almost constant.

After the NIPA/NIPMA ratios needed to obtain the required T_VPT_ were determined, the core/shell particles were synthesized using these ratios. Figure 3 shows a linear increase of T_VPT_ with increasing NIMPA concentration in the copolymer. M. Kokufuta, S. Sato, and E. Kokufuta [22] determined the LCST of NIPA-NIPMA copolymers as a function of composition and found that it increased linearly with increasing mole fraction of NIPAM in the copolymer. These authors used the values of 32 °C and 42 °C for the LCST of PNIPA and PNIPMA, respectively.

Figure 4 shows that the size of poly(NIPA-co-NIPMA)/CS core/shell nanoparticles made with monomer ratios similar to those used in the emulsion polymerization of NIPA/NIPMA turned out to be much smaller (compare Figure 2 and Figure 4). The smaller core/shell particles size is explained by the use of CS as the surfactant instead of Brij 58. CS can form micelles in acetic acid aqueous solutions above a critical concentration [23]. CMC values of CS were reported to range from 0.718 to 1.815 y g/L (the higher the pH, the larger the CMC [23]). In this work, we used a CS concentration of 10 g/L, a value that is well above the CMC at any pH. It is proposed that the polymerization occurs in the aqueous phase, and once the poly(NIPA-co-NIPAM) chains exceed their critical length, they introduce into the CS micelles. The absence of other size distributions in the QLS measurements (Figure 1) demonstrates that all the copolymer radicals formed in the aqueous phase penetrated the micelles’ interior. Figure 4 also shows that decreasing the NIPA/NIPMA ratio increases the T_VPT_ and shifts to slightly higher temperatures than those shown in Figure 3. The use of CS and acetic acid could cause the concentration of the monomers inside the micelles to be different from that obtained when Brij 58 was used and would explain the slightly higher T_VTP_ compared to those in Figure 2. However, since high conversions (above 95%) were obtained, the PNIPA/PNIPMA ratio will differ only slightly based on the monomer ratio used. Funduenau et al. [38], in the copolymerization of NIPA and NIPMA in solution, observed that the PNIPA/PNIPMA ratio in the copolymer was lower than in the feed, suggesting that NIPMA is more reactive than NIPA. 

The characteristic bands of PNIPA, PNIMPA, and CS were detected by FTIR, confirming their presence in the nanoparticles (Figure 5). 

The core/shell structure was proven by the increase in the size of the particles when the CS was crosslinked (Table 2) and by TEM (Figure 6) where a well-defined dark core and a thin, clearer shell can be observed in the particles. Because of the drying of the samples for TEM analysis, the particle size was smaller than that determined by QLS.

Figure 7 and Figure 8 show that at the two pHs studied, a higher amount of drug released and a higher release rate were obtained with the nanoparticles with T_VPT_ of 40 °C. This result is explained because the release experiments were performed at the T_VPT_ of the nanoparticles, so the higher the temperature, the higher the mobility of the drug molecules. Less drug was released at pH = 7.4 (compare Figure 7 and Figure 8); the lower amount of drug released is because chitosan is pH-sensitive and becomes hydrophobic at a pH above 6.4, causing its collapse, which hinders drug release. Moreover, at acidic pH, the drug is found as its salt (doxycycline hyclate), which is soluble in water; at alkaline pH, the salt is converted into doxycycline, which has low water solubility. Huang et al. reported a lower amount of drug released from NIPA/CS core/shell nanoparticles at alkaline pH than at acidic pH [23]. Alvarado et al. on the release of doxycycline hyclate from NIPA/CS core/shell nanocomposites also reported that at alkaline pH, the amount released was much lower than at acidic pH [26].

**Figure 10:**
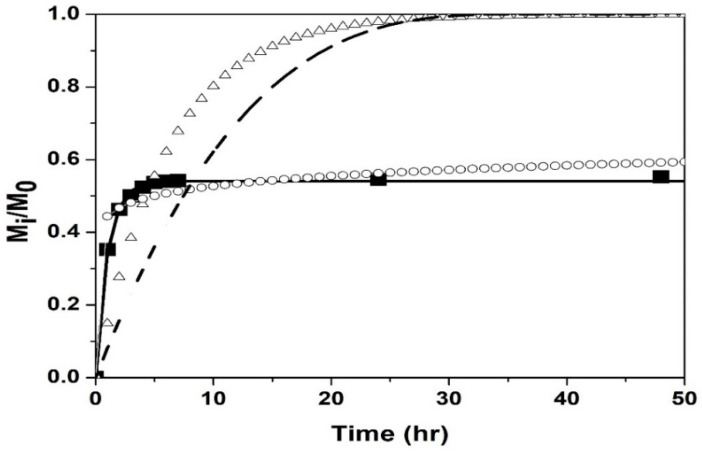
Comparison of the models with the experimental drug release data of nanohydrogels with T_VPT_ of 40 °C at pH = 7.4: Experimental (◼); Weibull (Δ); Korsmeyer–Peppas and extended Korsmeyer–Peppas (ο); first order (▬); Hixson-Crowell model (─ ─).

Figure 9 and Figure 10 and the coefficient of determination, R^2^, reported in Table 4, indicate that the first-order model best fits the experimental data and suggests that the release mechanism is diffusion-controlled by the concentration gradient. The Weibull and Hixson-Crowell models gave the worst results. The Korsmeyer–Peppas and extended Korsmeyer–Peppas models fitted the experimental data adequately up to 75% drug release. The parameter “*b*” is small and negative (Table 4), indicating that the bursting effect is not important so that there is no drug on the surface but that it is all inside the nanohydrogels. The fits of the Korsmeyer–Peppas and extended Korsmeyer–Peppas models are identical because the value of “*b*” is insignificant. 

**Figure 11:**
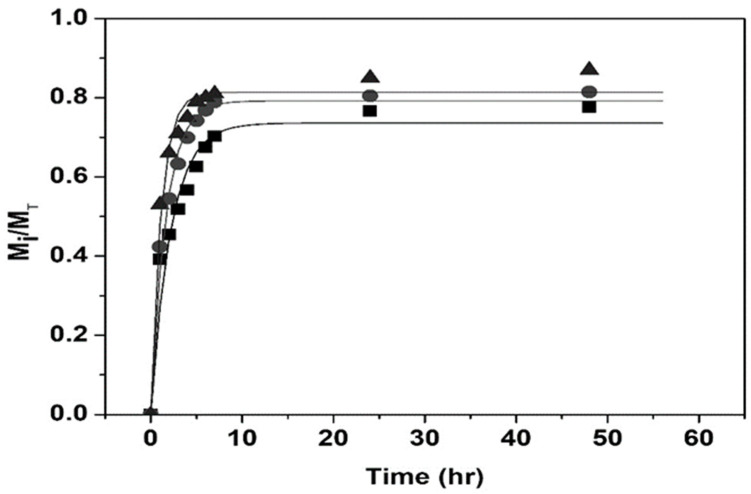
Comparison of experimental drug release data vs. first-order model at pH = 2.0; T_VPT_ of: 38 °C (◼); 39 °C (●); 40 °C (▲).

**Figure 12:**
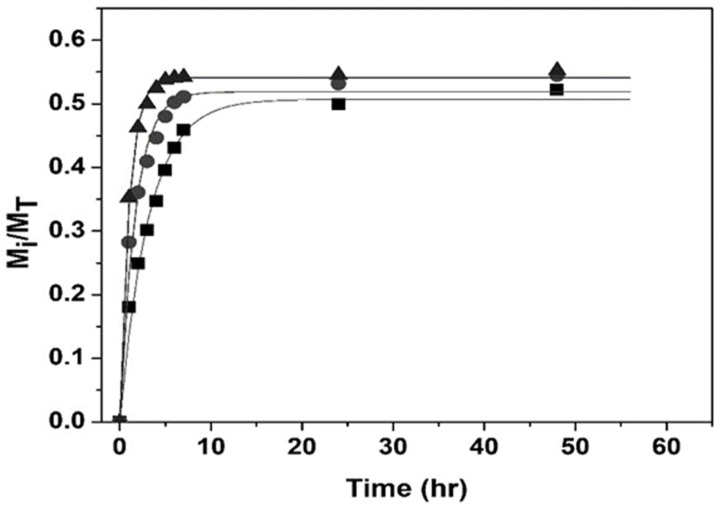
Comparison of experimental drug release data vs. first-order model at pH = 7.4; T_VPT_ of: 38 °C (◼); 39 °C (●); 40 °C (▲).

Figure 11 and Figure 12 show good agreement between the first-order model and experimental drug release data by nanohydrogels with T_VPT_ of 38, 39, and 40 °C at pH = 2.0 and 7.4.

In conclusion, we have synthesized pH- and temperature-sensitive poly(NIPA-co-NIPAM)/CS core/shell nanohydrogels by batch emulsion polymerization. These core/shell nanoparticles have a T_VPT_ of 38, 39, or 40 °C, the temperature present in the human body in response to infection, making them good candidates for use as drug delivery agents. At an alkaline pH, the drug has low water solubility, and the CS shell collapses making drug release difficult, which explains the lower amount released at this pH. The first-order model best fits the experimental drug release data, suggesting that the release mechanism is diffusion-controlled by the concentration gradient.

## Figures and Tables

**Table 1:** NIPMA/NIPA ratio and moles of monomers added to the reactor.

T_VPTc_ (°C)	NIPA Moles	NIPMA Moles	NIPMA/NIPA Ratio	NMBA Moles
38	0.014	0.011	0.785	0.12
39	0.012	0.013	1.083	0.12
40	0.010	0.014	1.400	0.12

**Table 2:** Conversion and particle size of core/shell nanohydrogels with T_VPT_ of 38, 39, and 40 °C. Sample 1 before crosslinking, Sample 2 after crosslinking.

T_VPT_ (°C)	Sample	Average Size (nm)	Conversion
38	1	296.5	96.1
	2	312.9	
39	1	300.8	97.7
	2	315.3	
40	1	300.1	96.4
	2	316.2	

**Table 3:** Drug content in nanohydrogels.

	T_VPT_ of the Nanohydrogels (°C)		
T_VPT_	38	39	40
% of drug content	5.00	5.20	5.41

**Table 4:** Fit parameters and determination coefficient, *R*^2^.

	pH = 2.0				pH = 7.4		
Model	*K*	*n*	*R^2^*	*b*	*k*	*n*	*R^2^*
$\frac{M_{i}}{M_{T}}=kt^{n}$	0.632	0.098	0.9647		0.444	0.0742	0.943
$\frac{M_{i}}{M_{T}}=b+kt^{n}$	0.632	0.098	0.9647	−0.001	0.445	0.7407	0.943
$\frac{M_{i}}{M_{T}}=1-e^{{\left(-\frac{t}{kw}\right)}^{n}}$	16.9	7.009	0.7872		26.16	4.242	0.00
$\frac{M_{i}}{M_{T}}=k\left(1-e^{-nt}\right)$	0.814	0.887	0.9801		0.541	0.998	0.988
$M_{T}^{1/3}-M_{R}^{1/3}=k_{H}\times t$	0.028		0.005		0.033		0.008

## Data Availability

Not applicable.
